# Evaluation of Two Chemiluminescent and Three ELISA Immunoassays for the Detection of SARS-CoV-2 IgG Antibodies: Implications for Disease Diagnosis and Patients’ Management

**DOI:** 10.3389/fimmu.2020.609242

**Published:** 2020-12-23

**Authors:** Matthaios Speletas, Maria A. Kyritsi, Alexandros Vontas, Aikaterini Theodoridou, Theofilos Chrysanthidis, Sophia Hatzianastasiou, Efthimia Petinaki, Christos Hadjichristodoulou

**Affiliations:** ^1^ Department of Immunology & Histocompatibility, Faculty of Medicine, University of Thessaly, Larissa, Greece; ^2^ Laboratory of Hygiene and Epidemiology, Faculty of Medicine, University of Thessaly, Larissa, Greece; ^3^ First Internal Medicine Department, Infectious Diseases Division, AHEPA Hospital, Medical School, Aristotle University of Thessaloniki, Thessaloniki, Greece; ^4^ National Public Health Organization, Athens, Greece; ^5^ Department of Microbiology, University Hospital of Larissa, University of Thessaly, Larissa, Greece

**Keywords:** COVID-19, immunoassay, IgG, ELISA, chemiluminescent

## Abstract

The estimation of anti-SARS-CoV-2 IgG antibodies is possibly the best approach to accurately establish the number of infected individuals and the seroprevalence of COVID-19 within a population. Thus, several commercial immunoassays have recently been developed. The purpose of our study was to assess the performance of five commonly used immunoassays in Greece (3 ELISA, namely Euroimmun SARS-CoV-2, GA GENERIC SARS-CoV-2 and Vircell COVID-19; and 2 chemiluminescent, namely ABBOTT SARS-CoV-2 and ROCHE Elecsys Anti-SARS-CoV-2 test) for the detection of anti-SARS-CoV-2 IgG antibodies. Sera specimens derived from 168 individuals were utilized to assess the specificity and sensitivity score of each assay. Among them, we included 99 COVID-19 patients (29 asymptomatic, 36 with symptom onset 4 to 14 days before serum sampling, and 34 with symptom initiation ≥ 15 days ago), and 69 volunteers with sera specimens collected prior to the SARS-CoV-2 outbreak and maintained at −80°C. We demonstrated that chemiluminescent immunoassays exhibit a significantly higher specificity score but a lower sensitivity, compared to ELISA immunoassays. Moreover, immunoassays detecting IgG antibodies against SARS-CoV-2 N protein instead of S protein alone are more reliable, considering both specificity and sensitivity scores. Interestingly, all asymptomatic patients displayed anti-SARS-CoV-2 IgG antibodies, confirmed by at least two immunoassays. We suggest that chemiluminescent assays could be used as screening methods for the detection of anti-SARS-CoV-2 antibodies to evaluate the possible prevalence of disease in the general population, while ELISA assays would be more reliable to evaluate, and follow-up confirmed COVID-19 patients.

## Introduction

As the coronavirus disease 2019 (COVID-19) pandemic continues to affect countries worldwide, the World Health Organization (WHO) is urging health authorities to rigorously test all suspected cases in order to isolate patients and interrupt the transmission chain (1). The gold standard method for diagnosis of COVID-19 is the detection of severe acute respiratory syndrome coronavirus 2 (SARS-CoV-2) genetic material with real-time PCR. However, several affected individuals never display symptoms of the disease, resulting in an underestimation of disease incidence and prevalence (2). Therefore, detection of anti-SARS-CoV-2 IgG antibodies is one of the better approaches available in order to determine the number of affected individuals in the community; the latter is clearly crucial for decision-making to inform public health policies.

Current studies have concluded IgG to be positive as early as the fourth day after symptom onset, although higher levels of IgG occur during the second and third week of COVID-19 (3, 4). Knowledge surrounding antibody tests for the detection of SARS-CoV-2 antibodies is still evolving; thus, the evaluation of commercial kits is critical. Tests that detect antibodies to nucleocapsid (N) antigen are expected to be more sensitive since the majority of antibodies are produced against the most abundant protein of the virus, which is the N protein (5). On the other hand, antibodies to the receptor-binding domain of spike glycoprotein (RBD-S) would be more specific, since RBD-S is the host attachment protein, and these have been correlated with the severity of the disease (5, 6).

Traditionally, antibody determination is performed using various techniques such as Enzyme-Linked ImmunoSorbent Assay (ELISA), chemiluminescent immunoassay (CLIA), rapid lateral flow (immunochromatographic) tests or fluorescence Immunoassays (FIA). ELISA and variations of CLIA are the most reliable solutions, particularly for COVID-19 (7–9).

The purpose of the current study was to assess the performance of three ELISA and two chemiluminescent assays that are commonly used in Greece, regarding sensitivity and specificity in detecting IgG anti-SARS-CoV-2 antibodies.

## Materials and Methods

### Study Design and Commercial Tests Validated

*Serum samples from COVID-19 confirmed cases*: A total of 99 serum samples were collected from April to May; fifty-seven samples originated from patients on a cruise ferry during a COVID-19 outbreak investigation with an attack rate of 31.3% (119/380 travelers). The remaining 42 samples were derived from hospitalized patients in both a reference hospital (AHEPA Hospital, Thessaloniki, Greece) and a medical unit for the isolation of patients to limit disease transmission (AROGI, Larissa, Greece). All patients displayed real-time PCR confirmed COVID-19, performed using a nasopharyngeal swab. The patients were further divided into three groups according to symptom onset, as follows:

Group A: 29 patients without symptoms at the time of serum collection; for a large majority of patients (24 from the cruise ferry) the serum sampling and the nasopharyngeal swab were taken the same day, while for the remaining patients this was done 4 to 10 days after PCR positivity

Group B: 36 patients with symptom onset 4 to 14 days prior to serum sampling,

Group C: 34 patients with symptom initiation ≥ 15 days ago.

*Serum samples for specificity evaluation*: A total of 69 serum samples were used, which were derived from a seroprevalence study on West Niele virus infection (10), performed on 2013 and maintained at −80°C.

The five evaluated immunoassays included:

1. The ABBOTT SARS-CoV-2 IgG assay (Abbott, Illinois, U.S.A.), which is a chemiluminescent microparticle immunoassay (CMIA) for the qualitative detection of IgG antibodies that target the N virus protein. The calculated S/Co values of <1.4 were reported as negative, whereas ≥ 1.4 were reported as positive. Tests were performed in the high-throughput ARCHITECT i2000SR.
2. The ROCHE Elecsys Anti-SARS-CoV-2 serology test (La Roche Ltd, Basel, Switzerland), which is an electrochemiluminescence immunoassay (ECLIA), is similarly used for the detection of IgG antibodies against N antigen. When the reactions were completed, S/Co values of < 1.0 were reported as negative, whereas ≥ 1.0 were reported as positive; the Cobas 8000 immunoassay analyzer was used.
3. The Euroimmun SARS-CoV-2 IgG (Euroimmun, Lübeck, Germany). The test performs specific detection of IgG against SARS-CoV-2 using the S1 domain of the S protein, including the immunologically relevant receptor binding domain (RBD). The ratio interpretation was as follows: < 0.8 negative, ≥ 0.8 to < 1.1 borderline and ≥ 1.1 positive. The assays were performed in the Euroimmun Analyzer I.
4. The GA-GENERIC CoV-2 IgG (GA GENERIC ASSAYS GmbH, Dahlewitz, Germany). This Elisa kit detects IgG antibodies against S and N antigens of SARS-CoV-2 (recombinant) different strains. The binding index was calculated by the ratio of OD values of samples to the cutoff, and samples were characterized as positive, negative and ambiguous when the BI was ≥ 1.1, < 0.9, and 0.9 to 1.1, respectively. The assay was performed in the DYNEX analyzer.
5. The Vircell COVID-19 ELISA IgG (Vircell Spain S.L.U., Granada, Spain). The test uses recombinant antigen from both S and N proteins. Results were expressed as the ratio of (sample OD/cutoff serum mean OD) x 10 and using this equivalent calculation the index value thresholds for positive, negative and ambiguous results were ≥ 6.0, <4.0, and 4.0 to 6.0, respectively. The Elisa assay was performed manually according to the manufacturer’s instructions.

All tests were performed and interpreted according to the manufacturer’s instructions for each immunoassay respectively, in a biosafety level (BSL)-2 capacity laboratory. While all sera samples were analyzed for the first 4 immunoassays, the Vircell COVID-19 ELISA IgG assay was performed in only 70 patients and 41 volunteers’ samples, due to a depletion of serum stocks. Details regarding the attributes for the evaluated anti-SARS-CoV-2 IgG serologic assays are presented in **Table 1**.

**Table 1:** The anti-SARS-CoV-2 immunoassays evaluated in this study.

Characteristics	ABBOTT SARS-CoV-2 IgG assay	ROCHE Elecsys Anti-SARS-CoV-2 serology	Euroimmun SARS-CoV-2 IgG assay	GA GENERIC CoV-2 IgG assay	Vircell COVID-19 ELISA IgG assay
Test principle	CMIA	ECLIA	ELISA	ELISA	ELISA
Antigen	Nucleocapsid	Nucleocapsid	Spike S1	Nucleocapsid and spike	Nucleocapsid and spike
Sample type	Serum, plasma	Serum, plasma	Serum	Serum	Serum
Sample volume	25 μl	20 μl	10 μl	10 μl	20 μl
Result calculation	Index (S/Co)	Index (S/Co)	Index (S/Co)	Index (S/Co)	Index (S/Co)
Positive cutoff index	≥ 1.4	≥ 1.0	≥ 1.1	≥ 1.1	≥ 6.0
Gray zone cutoff index	NA	NA	0.8–1.1	0.9–1.1	4.0–6.0

All participants (patients and blood volunteers) provided informed consent and the study was approved by the ethical committee of the Faculty of Medicine, University of Thessaly (No. 2116).

### Statistical Analysis

Descriptive statistics were used to describe the study variables. Proportions and frequency were reported for the categorical variables. Sensitivity (Se) and specificity (Sp) were estimated with 95% Confidence Intervals (CI), based on binomial distribution (11). Se and Sp were analyzed with the use of Chi-square tests (12). A 5% significance level was set for all the analyses. Statistical analysis was carried out using Microsoft Excel and SPSS (version 25.0).

## Results

**Figure 1:**
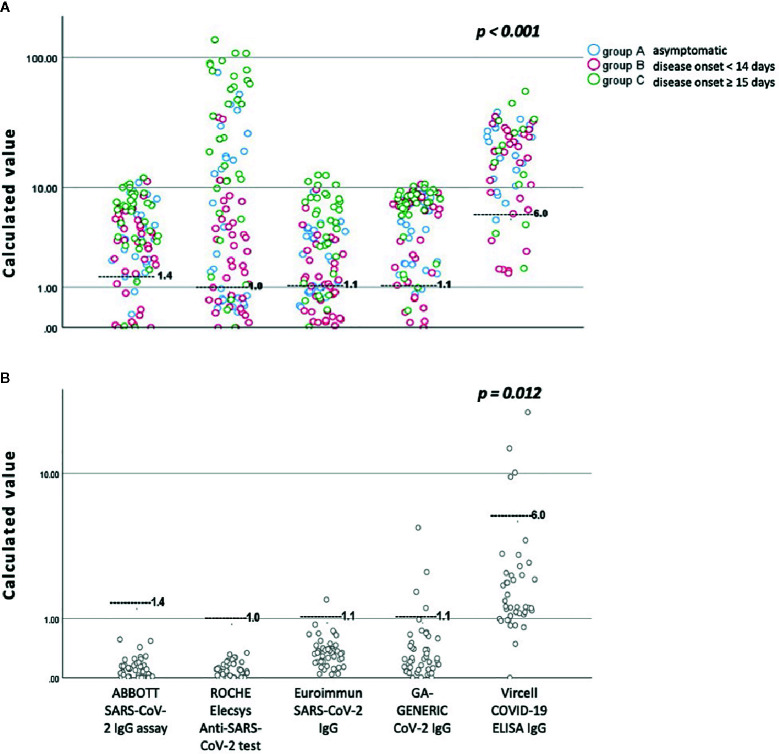
An overview of anti-SARS-CoV-2 IgG antibodies detected by all assays evaluated in this study, according to the day of blood sampling from the day of disease onset. **(A)** Sera specimens from patients with confirmed COVID-19 disease. **(B)** Sera specimens from volunteers collected before the SARS-CoV-2 outbreak. The black gray line represents the cutoff for positivity of each immunoassay. For a detailed presentation of the results, the reader is referred to **Tables 2** and **3** and the printed version of this manuscript.

**Figure 1** presents an overview of anti-SARS-CoV-2 IgG antibodies detected in the sera specimens of study patients and volunteers, considering that all evaluated assays are qualitative and/or semi-quantitative, as clarified by their instruction manuals.

Interestingly, both the ABBOTT SARS-CoV-2 IgG assay and ROCHE Elecsys Anti-SARS-CoV-2 serology test showed specificity of 100% (95% CI: 94.8-100%) since there were no false positive results recorded in the pre-COVID-19 group. The Sp score for Euroimmun SARS-CoV-2 IgG test was calculated at 97.1% (95% CI: 89.9-99.6%), for the GA GENERIC CoV-2 IgG at 92.7% (95% CI: 83.9-97.6%), and finally for the Vircell COVID-19 ELISA IgG test at 90.2% (95% CI: 76.9–97.3%). As presented in detail in **Table 2**, differences between the Sp scores of the evaluated immunoassays were found to be statistically significant (p = 0.012).

**Table 2:** Specificity of anti-SARS-CoV-2 immunoassays evaluated in this study.

Parameters	ABBOTT SARS-CoV-2 IgG assay	ROCHE Elecsys Anti-SARS-CoV-2	Euroimmun SARS-CoV-2 IgG	GA GENERIC CoV-2 IgG assay	Vircell COVID-19 ELISA IgG assay	Chi-Square test (sig)
No sera analyzed	69	69	69	69	41	
Negative (no, %)	69 (100)	69 (100)	67 (97.1)	64 (92.8)	37 (90.2)	
Ambiguous (no, %)	0 (0)	0 (0)	1 (1.4)	2 (2.9)	0 (0)	
Positive (no, %)	0 (0)	0 (0)	1 (1.4)	3 (4.3)	4 (9.8)	
Specificity with 95%CI (%)	100 (94.8–100)	100 (94.8–100)	97.1 (89.9–99.6)	92.7 (83.9–97.6)	90.2 (76.9–97.3)	**p = 0.012**

CI, confidence interval. The statistically significant values are presented as bold.

**Figure 2:**
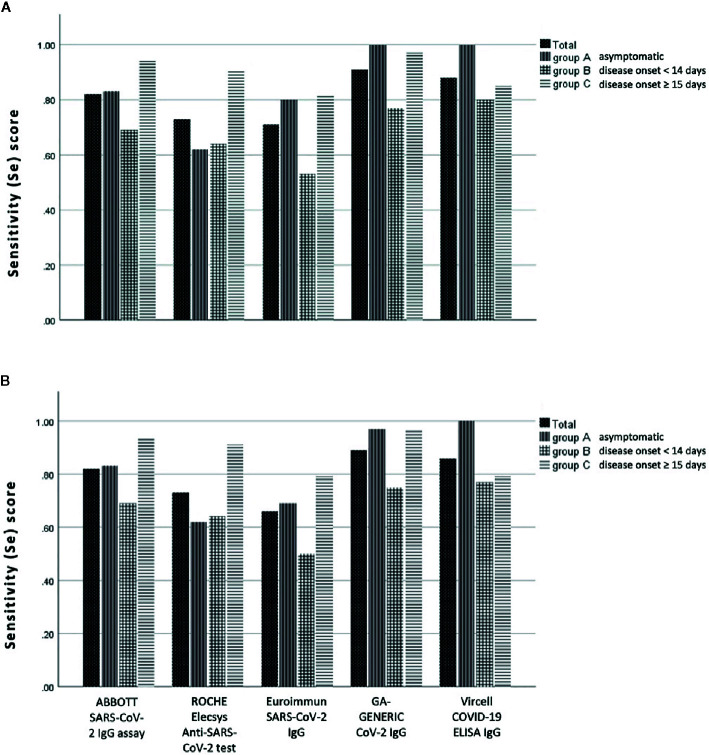
Sensitivity (Se) scores of anti-SARS-CoV-2 IgG assays evaluated in this study, according to the day of blood sampling from the day of disease onset: **(A)** with and **(B)** without the ambiguous (gray zone) samples.

Concerning the overall Se, ABBOTT SARS-CoV-2 IgG assay demonstrated a score of 81.8% (95% CI: 72.8–88.9%), ROCHE Elecsys Anti-SARS-CoV-2 serology test a score of 72.7% (95% CI: 62.9–81.2%), Euroimmun SARS-CoV-2 IgG a score of 65.7% (95% CI: 55.4–74.9%), GA GENERIC CoV-2 IgG a score of 88.9% (95% CI: 80.99–94.32%) and Vircell COVID-19 ELISA IgG a score of 85.7% (95% CI: 75.3–92.9%),with the difference also statistically significant (p < 0.001). The same difference was not documented within the group of patients that had disease onset of more than 15 days from blood sampling (p = 0.225). **Table 3** and **Figure 2** also present detailed data and the estimated Se of each immunoassay, including ambiguous samples as well as patients’ sera for all patient groups (asymptomatic, less and greater than 14 days of a documented disease onset).

**Table 3:** Sensitivity of anti-SARS-CoV-2 immunoassays evaluated in this study.

Parameters	ABBOTT SARS-CoV-2 IgG assay	ROCHE Elecsys Anti-SARS-CoV-2	Euroimmun SARS-CoV-2 IgG	GA GENERIC CoV-2 IgG assay	Vircell COVID-19 ELISA IgG assay	Chi-square test (sig.)
**Total**—No sera analyzed	99	99	99	99	70	
Positive (no, %)	81 (81.8)	72 (72.7)	65 (65.7)	88 (88.9)	60 (85.7)	
Ambiguous (no, %)	0 (0)	0 (0)	7 (7.0)	2 (2.0)	2 (2.9)	
Negative (no, %)	18 (18.2)	27 (27.3)	27 (27.3)	9 (9.1)	8 (11.4)	
Sensitivity with 95%CI (%)	81.8 (72.8–88.9)	72.7 (62.9–81.2)	65.7 (55.4–74.9)	88.9 (81.0–94.3)	85.7 (75.3–92.9)	**p < 0.001**
Sensitivity with 95%CI (%) ^	as above	as above	70.7 (62.9–81.2)	90.9 (81.0–94.3)	88.2 (78.1–94.9)	**p = 0.001**
**Group A: Asymptomatic COVID-19 patients**						
No sera analyzed	29	29	29	29	25	
Positive (no, %)	26 (89.7)	23 (79.3)	20 (68.9)	28 (96.6)	25 (100)	
Ambiguous (no, %)	0 (0)	0 (0)	4 (13.8)	1 (2.4)	0 (0)	
Negative (no, %)	3 (10.3)	6 (20.7)	5 (26.3)	0 (0)	0 (0)	
Sensitivity with 95%CI (%)	82.8 (64.2–94.2)	62.1 (42.3–79.3)	69.0 (49.2–84.7)	96.6 (82.2–99.9)	100 (86.3–100)	**p = 0.021**
Sensitivity with 95%CI (%) ^	as above	as above	80.0 (59.3–93.2)	100 (87.7–100)	as above	p = 0.087
**Group B: COVID-19 patients (symptoms onset < 15 days)**						
No sera analyzed	36	36	36	36	31	
Positive (no, %)	25 (69.4)	23 (63.9)	18 (50.0)	27 (75.0)	24 (77.4)	
Ambiguous (no, %)	0 (0)	0 (0)	2 (5.6)	1 (2.8)	1 (3.2)	
Negative (no, %)	11 (30.6)	13 (36.1)	16 (44.4)	8 (22.2)	6 (19.4)	
Sensitivity with 95%CI (%)	69.4 (51.9–83.7)	63.9 (46.2–79.2)	50.0 (32.9–67.1)	75.0 (57.8–87.9)	77.4 (58.9–90.4)	p = 0.111
Sensitivity with 95%CI (%) ^	as above	as above	52.9 (35.1–70.2)	77.1 (59.9–89.6)	80.0 (61.4–92.3)	p = 0.125
**Group C: COVID-19 patients (symptoms onset ≥ 15 days)**						
No sera analyzed	34	34	34	34	14	
Positive (no, %)	32 (94.1)	31 (91.2)	27 (79.4)	33 (97.1)	11 (78.6)	
Ambiguous (no, %)	0 (0)	0 (0)	1 (2.9)	0 (0)	1 (7.1)	
Negative (no, %)	2 (5.9)	3 (8.8)	6 (17.7)	1 (2.9)	2 (14.3)	
Sensitivity with 95%CI (%)	94.1 (80.3–99.3)	91.2 (76.3–98.1)	79.4 (62.1–93.0)	97.1 (84.7–99.9)	78.6 (49.2–95.3)	p = 0.082
Sensitivity with 95%CI (%) ^	as above	as above	81.8 (64.5–93.0)	as above	85.7 (57.2–98.2)	p = 0.225

CI, confidence interval.

^In this case the sensitivity estimated including ambiguous samples. The statistically significant values are presented as bold.

**Figure 3:**
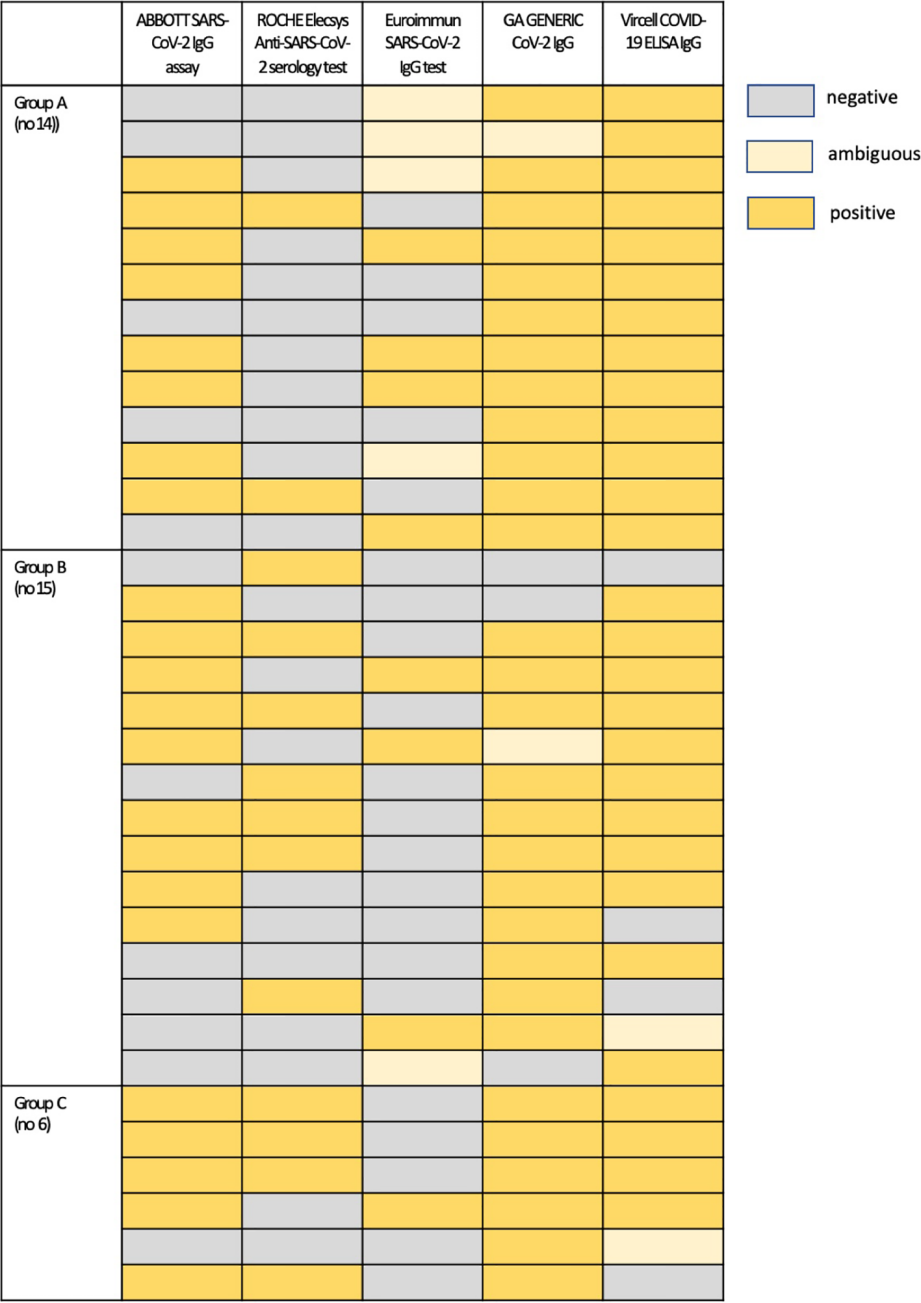
An overview of antibody negativity and positivity according to the evaluated assay in patients who were not antibody positive with all assays: Group A, asymptomatic patients; Group B, patients with symptom onset 4 to 14 days prior to serum sampling; Group C, patients with symptom onset ≥ 15 days prior to serum sampling.

Thirty-one (31) out of 99 patients (31.3%) were not antibody positive with all assays despite being SARS-CoV-2 PCR positive. **Figure 3** presents an overview of the pattern of antibody negativity according to the evaluated assay in these patients. Strikingly, all asymptomatic COVID-19 individuals displayed IgG antibodies in their sera, confirmed by at least two immunoassays. Moreover, sera samples from 4 patients with a disease onset of <14 days and one of >15 days were found to be negative with all assays; however, all these patients were found positive in new sera obtained 2 to 3 weeks later, as determined by either ABBOTT SARS-CoV-2 IgG assay (3 samples) or by GA GENERIC CoV-2 IgG ELISA assay (2 samples) (**Supplementary Table 1**). Finally, there was one false-positive case established by one immunoassay, which was also found to be positive by another one. There was only one sample from a volunteer that had an ambiguous result with both Euroimmun SARS-CoV-2 IgG and GA GENERIC CoV-2 IgG ELISA immunoassays.

## Discussion

In this study we evaluated the Sp and Se of five commonly used immunoassays for the detection of anti-SARS-CoV-2 IgG antibodies. As illustrated in **Tables 2** and **3** and **Figure 2**, both chemiluminescent assays exhibit a significantly higher Sp score than ELISA assays, while 2 out of 3 ELISA assays (GA GENENIC and Vircell) display a significantly higher Se score than chemiluminescent ones.

A possible explanation of our results could be the fact that the evaluated assays detect different antigen components. Subsequently, antibody responses against each aforementioned antigen may develop at different times. Thus, immunoassays detecting IgG antibodies against N protein were found to be more sensitive than Euroimmun, which recognizes antibodies against the S1 domain of the SARS-CoV-2 S protein. In this context and as expected, the sensitivity of all assays was higher when sera samples were derived from patients whose symptom onset was greater than 15 days from blood sampling (**Table 3**, **Figure 1**).

Our results were in accordance with previous studies on other coronaviruses showing a significantly higher sensitivity of antibody tests based on the N protein (13, 14). This could be attributed to the fact that the majority of antibodies are raised against the N protein, while antibodies against the S protein are considered more specific and associated with a neutralizing capacity (15, 16). It is worth noting that recent studies showed a decline in the IgG antibodies against the N protein over time, while the response to the S protein was proven to be more stable (17).

Moreover, the instruction manual of the ABBOTT SARS-CoV-2 IgG assay provides results rather similar to ours for Sp (96.63% with 95% CI 95.05–99.90). However, Se is somewhat overestimated for samples of >14 days with Se of 100% (95% CI 95.89–100.00), a finding that was not confirmed in our study. Similarly, we could not confirm the 100% Se of ROCHE Elecsys Anti-SARS-CoV-2 serology test for samples of >14 days from disease onset, although Sp of the assay was very high and the Se score reached > 90% for samples with a disease onset of ≥ 15 days (**Table 3**, **Figure 2**). Conversely, GA GENERIC CoV-2 and Vircell COVID-19 ELISA assays were determined to be more sensitive than chemiluminescent assays, and their Se scores were rather equivalent to those presented within their instruction manuals for samples of > 14 days from disease onset (97.1% vs 98% and 86.7% vs 85%, respectively). However, Sp scores of both assays were lower than expected (92.7% vs 98.0% and 90.2% vs 98.0%, respectively).

We observed that the Euroimmun SARS-CoV-2 IgG assay exhibited the lower Se score compared to other assays. This is in accordance with previous studies indicating that the Se score of this assay is rather low; thus, in the study by Montesinos et al. the Sp and Se scores of the assay were almost similar to our study (98.6% vs 97.1%, and 68.1% vs 65.7%, respectively) (8), while in the study of Lippi et al. the Se score was even lower (38.9% for patients with a disease onset of ≥ 5 days) (18). Likewise, Kohmer et al. demonstrated that the Se for samples with a disease onset of 5 to 9 days and 10 to 18 days was 70.6% and 100% for Vircell COVID-19 ELISA, and 58.8% and 93.8% for Euroimmun SARS-CoV-2 IgG, respectively (19). On the other hand, we could not confirm the higher Se score of the ABBOTT SARS-CoV-2 IgG assay that was reported by Meschi et al (20)., although this automated serological assay was demonstrated along with ROCHE Elecsys Anti-SARS-CoV-2 serology test, as more specific compared to ELISA assays, and we recommend them in cases of high testing loads.

Interestingly enough we demonstrated that all asymptomatic COVID-19 patients were found positive for anti-SARS-CoV-2 IgG antibodies. This finding further supports the notion that measurement of IgG antibodies is one of the most reliable tools to clarify the true prevalence of COVID-19 within the community. However, possible cross‐reactivity for antibodies against endemic coronaviruses (e.g. HCoV-OC43 and HCoV-229E) or other active infectious diseases (e.g. EBV or CMV), as described by recent studies (19, 21), should always be taken into consideration.

A possible limitation of our study could be the fairly low number of samples evaluated. Nevertheless, we consider that the number of analyzed sera was adequate for validation of the assays and provided valuable results. Based on our findings we further evaluated the ABBOTT SARS-CoV-2 IgG assay in a cohort of 305 negative samples (before the evolution of SARS-CoV-2) and we obtained similar results (Sp 99.7% with 95% CI: 98.2–100%). These results have been presented in a recent manuscript estimating the prevalence of IgG antibodies in Greece during March and April 2020 (22).

Based on the aforementioned findings, we conclude that: 1) immunoassays detecting IgG antibodies against SARS-CoV-2 N protein instead of S protein alone are more reliable, considering Sp and Se scores, and 2) chemiluminescent assays could be recommended as screening methods for the detection of anti-SARS-CoV-2 IgG antibodies in the general population (particularly when the expected seroprevalence is low), while ELISA assays are more reliable for the evaluation and follow-up of confirmed COVID-19 patients.

## Data Availability Statement

The raw data supporting the conclusions of this article will be made available by the authors, without undue reservation.

## Ethics Statement

The study was approved by the ethical committee of the Faculty of Medicine, University of Thessaly (No. 2116). The patients/participants provided their written informed consent to participate in this study. 

## The COVID-19 GEnomics and SErology (COGESE) study group

The following Authors, who are listed in alphabetical order, contributed to the work of the COVID-19 GEnomics and SErology (COGESE) study group:

**Lemonia Anagnostopoulou**, Laboratory of Hygiene and Epidemiology, Faculty of Medicine, University of Thessaly, Larissa, Greece; **Katerina Dadouli**, Laboratory of Hygiene and Epidemiology, Faculty of Medicine, University of Thessaly, Larissa, Greece; **Georgios Germanidis**, First Internal Medicine Department, Infectious Diseases Division, AHEPA Hospital, Medical School, Aristotle University of Thessaloniki, Greece; **Panagiotis Kollaras**, First Internal Medicine Department, Infectious Diseases Division, AHEPA Hospital, Medical School, Aristotle University of Thessaloniki, Greece; **Symeon Metallidis**, First Internal Medicine Department, Infectious Diseases Division, AHEPA Hospital, Medical School, Aristotle University of Thessaloniki, Greece; **Paraskevi Mina**, Laboratory of Hygiene and Epidemiology, Faculty of Medicine, University of Thessaly, Larissa, Greece; **Varvara A. Mouchtouri**, Laboratory of Hygiene and Epidemiology, Faculty of Medicine, University of Thessaly, Larissa, Greece; **Dimitrios J. Nikoulis**, Laboratory of Hygiene and Epidemiology, Faculty of Medicine, University of Thessaly, Larissa, Greece; **Georgia Nikolopoulou**, National Public Health Organization, Athens, Greece; **Maria Tseroni**, National Public Health Organization, Athens, Greece; **Gerasimina Tsinti**, Department of Immunology & Histocompatibility, Faculty of Medicine, University of Thessaly, Larissa, Greece.

## Author Contributions

MS and CH conceived and designed the study. MK, AV, and AT conducted the experiments. TC and SH critically revised the manuscript for important intellectual content. MS, MK, AT, AV, TC, SH, and the remaining members of the COGESE study group acquired both patients’ and volunteers’ sera and experimental data. MS, MK, and CH wrote the manuscript. All co-authors approved the final version. All authors contributed to the article and approved the submitted version.

## Conflict of Interest

The authors declare that the research was conducted in the absence of any commercial or financial relationships that could be construed as a potential conflict of interest.
