# Supplementary material for: Evaluation of Two Chemiluminescent and Three ELISA Immunoassays for the Detection of SARS-CoV-2 IgG Antibodies: Implications for Disease Diagnosis and Patients’ Management
Source: Front Immunol. 2020 Dec 23;11:609242. doi: 10.3389/fimmu.2020.609242 (PMC7785794; doi:10.3389/fimmu.2020.609242)
Supplement: Supplementary file 1 [file Table_1.docx]

**Supplementary Table 1**. Longitudinal titer of anti-SARS-CoV-2 antibodies in patients with an initial negative result.

|  | Day of 1^st^ serum sampling * | Assay | Ab titer | Day of 2^nd^ sampling * | Ab titer |
| --- | --- | --- | --- | --- | --- |
| Patient #B26 | 11 | GA GENERIC CoV-2 IgG assay **^^^** | 0.367 | 30 | 7.298 |
| Patient #B30 | 6 | ABBOTT SARS-CoV-2 IgG assay **^#^** | 0.068 | 28 | >8.00 |
| Patient #B31 | 6 | ABBOTT SARS-CoV-2 IgG assay **^#^** | 0.13 | 28 | 3.81 |
| Patient #B32 | 8 | ABBOTT SARS-CoV-2 IgG assay **^#^** | 0.02 | 21 | 5.85 |
| Patient #C16 | 15 | GA GENERIC CoV-2 IgG assay **^^^** | 0.292 | 37 | 1.335 |

**^^^** the positive cut-off index is ≥ 1.1, and the grey zone cut-off index is 0.8-1.1

**^#^** the positive cut-off index is ≥ 1.4
